# Potential Biomarkers to Predict Acute Ischemic Stroke in Type 2 Diabetes

**DOI:** 10.3389/fmolb.2021.744459

**Published:** 2021-12-01

**Authors:** Abu Saleh Md Moin, Manjula Nandakumar, Ahmed Al-Qaissi, Thozhukat Sathyapalan, Stephen L. Atkin, Alexandra E. Butler

**Affiliations:** ^1^ Diabetes Research Center (DRC), Qatar Biomedical Research Institute (QBRI), Hamad Bin Khalifa University (HBKU), Qatar Foundation (QF), Doha, Qatar; ^2^ Academic Endocrinology, Diabetes and Metabolism, Hull York Medical School, Heslington, United Kingdom; ^3^ Leeds Medical School, Leeds, United Kingdom; ^4^ Department of Research, Royal College of Surgeons of Ireland, Al Muharraq, Bahrain

**Keywords:** type 2 diabetes, endothelial proteins, biomarkers, ischemic stroke, predictive biomarkers

## Abstract

**Background and Purpose:** Patients with type 2 diabetes (T2D) have increased risk of cardiovascular disease (CVD), encompassing myocardial infarction, stroke, and peripheral vascular disease. We hypothesized that those biomarkers indicative of acute ischemic stroke (AIS) seen in large vessel occlusion (LVO) may also be elevated in T2D and further enhanced by stress conditions; therefore, these proteins represent potentially predictive biomarkers for those T2D patients at high risk of AIS.

**Methods:** We performed an exploratory proteomic analysis in control subjects (*n* = 23) versus those with type 2 diabetes (T2D) (*n* = 23) who underwent a hyperinsulinemic clamp study to transient severe hypoglycemia [blood glucose <2.0 mmol/L (36 mg/dl)] in a prospective case-control study. We compared these proteins described as diagnostic and prognostic biomarkers for AIS due to LVO: lymphatic vessel endothelial hyaluronic acid receptor-1 (LYVE1), thrombospondin-1 (THBS1), pro-platelet basic protein (PPBP), and cadherin 1 (CDH1).

**Results:** At baseline (BL), PPBP (*p* < 0.05), THBS1 (*p* < 0.05), and CDH1 (*p* < 0.01) were elevated in T2D; LYVE1 was not different between controls and T2D subjects at BL or at subsequent timepoints. PPBP and THBS1 tended to increase at hypoglycemia in both cohorts, though reached significance only in controls (*p* < 0.05), returning to BL levels post-hypoglycemia. CDH1 levels were higher in T2D at BL, at hypoglycemia and up to 2-h posthypoglycemia, thereafter reverting to BL levels.

**Conclusion:** Elevated levels of PPBP, THBS1, and CDH1, circulatory proteins suggested as biomarkers of AIS due to LVO, may, in T2D patients, be prognostically indicative of a cohort of T2D patients at increased risk of ischaemic stroke. Prospective studies are needed to determine if this reflects future clinical risk.

Clinical trial reg. no: NCT03102801.

## Introduction

Patients with type 2 diabetes (T2D) have a well-documented 2-3-fold increased risk for cardiovascular disease (CVD), encompassing myocardial infarction, stroke, and peripheral vascular disease (Einarson et al., 2018), with ∼80% of mortality in T2D due to underlying CVD (Morrish et al., 2001). Approximately one-third of stroke patients have diabetes (Lau et al., 2019), with a higher prevalence reported in ischemic versus hemorrhagic stroke patients (Tsai et al., 2016).

In a recent publication, proteomic analysis of plasma samples was undertaken on patients with acute ischemic stroke (AIS) caused by large vessel occlusion (LVO) to identify a panel of diagnostic and prognostic biomarkers (Qin et al., 2019). Seven differentially expressed proteins were identified: lymphatic vessel endothelial hyaluronic acid receptor-1 (LYVE1), thrombospondin-1 (THBS1), pro-platelet basic protein (PPBP), secreted phosphoprotein-2 (SPP2), insulin-like growth factor-2 (IGF2), cadherin 1 (CDH1), and apolipoprotein C 4-2 (APOC4-APOC2), four of which were elevated (LYVE1, THBS1, PPBP, and IGF2) and subsequently validated by Western blot analysis in patients with AIS caused by LVO versus healthy control subjects. PPBP, THBS1, and LYVE1 are involved in endothelial function and hemostasis (Yamauchi et al., 2007; Stapor et al., 2013; Maneerat et al., 2017). PPBP is released from activated platelets, directs leukocytes to vascular injury sites (Ghasemzadeh et al., 2013) and is involved in ischemia-related inflammation (Ehlken et al., 2015; Maneerat et al., 2017). In central retinal vein ischemic occlusion, PPBP serves as an inflammatory biomarker (Ehlken et al., 2015) and plays a role in carotid atherosclerosis, inflammation and plaque instability leading to embolism (Perisic et al., 2016); however, PPBP, whilst shown to be elevated in AIS, did not appear to have any predictive value (Qin et al., 2019). THBS1 regulates angiogenesis (Yamauchi et al., 2007) and elevations in plasma levels have been reported in patients with ischemic stroke (Gao et al., 2015). THBS1was shown to be elevated in AIS and had positive predictive value at 3-months prognosis (Qin et al., 2019). LYVE1 is a vascular endothelial membrane receptor present in the lymphatic system; via interactions between lymphatic and blood endothelial cells, LYVE1 promotes angiogenesis (Stapor et al., 2013). In response to cerebrovascular injury in zebrafish, LYVE1 promotes lymphatic growth into brain parenchyma to guide vascular regeneration (Chen et al., 2019). LYVE1 was elevated in AIS and had positive predictive value at 3-month prognosis (Qin et al., 2019). CDH1 (or E-cadherin), a member of the cadherin superfamily, is a calcium-dependent cell adhesion protein that functions as a tumor suppressor (Yu et al., 2019). CDH1 is important in blood-brain barrier (BBB) function (Pal et al., 1997); ischemic stress may impact endothelial cell calcium flux, thereby altering the structure and function of the BBB (Abbruscato and Davis, 1999); CDH1 was noted to be downregulated in AIS but was not found to have any predictive value (Qin et al., 2019).

We hypothesized that these potential biomarkers of stroke may be enhanced in T2D, particularly under stress conditions such as hypoglycemia. In patients with T2D, these preidentified proteins, previously found to be predictive for AIS due to LVO, may therefore serve as biomarkers for AIS. Therefore, we performed proteomic analysis in control subjects versus those with type 2 diabetes (T2D), all of whom underwent transient severe hypoglycemia, to compare these proteins described as diagnostic and prognostic biomarkers for AIS due to LVO (Qin et al., 2019).

## Methods

Type 2 diabetes (T2D) (*n* = 23) and control (*n* = 23) subjects were enrolled in a prospective interventional case-controlled study performed in the Diabetes Centre at Hull Royal Infirmary (Clinical trial reg. no: NCT03102801; registration date April 06, 2017; start date March 01, 2017, end date January 10, 2018). All study participants signed an informed consent form prior to participation. The trial was approved by the North West-Greater Manchester East Research Ethics Committee (REC number: 16/NW/0518) and conducted according to the Declaration of Helsinki.

### Study Participants

All participants were Caucasian, aged 40–70 years. The T2D group had been diagnosed for <10 years; all were on a stable dose of medication (metformin, statin and/or angiotensin-converting enzyme inhibitor/angiotensin receptor blocker) for the preceding 3 months (Al-Qaissi et al., 2019). T2D subjects were excluded if on any anti-glycemic medication other than metformin or with poor glycemic control [HbA1c levels ≥10% (86 mmol/mol)]. Control subjects were excluded if diagnosed with type 1 or 2 diabetes or if HbA1c levels were >6% (42 mmol/mol). The following exclusion criteria were applied for both groups: current smokers, body mass index (BMI) < 18 or >50 kg/m^2^, excessive alcohol consumption, renal or liver disease, history or presence of malignant neoplasms within the last 5-years, diagnosis of psychiatric illness, history of pancreatitis or gastrointestinal tract surgery.

### Hyperinsulinemic Euglycemic Clamp Studies

**FIGURE 1:**
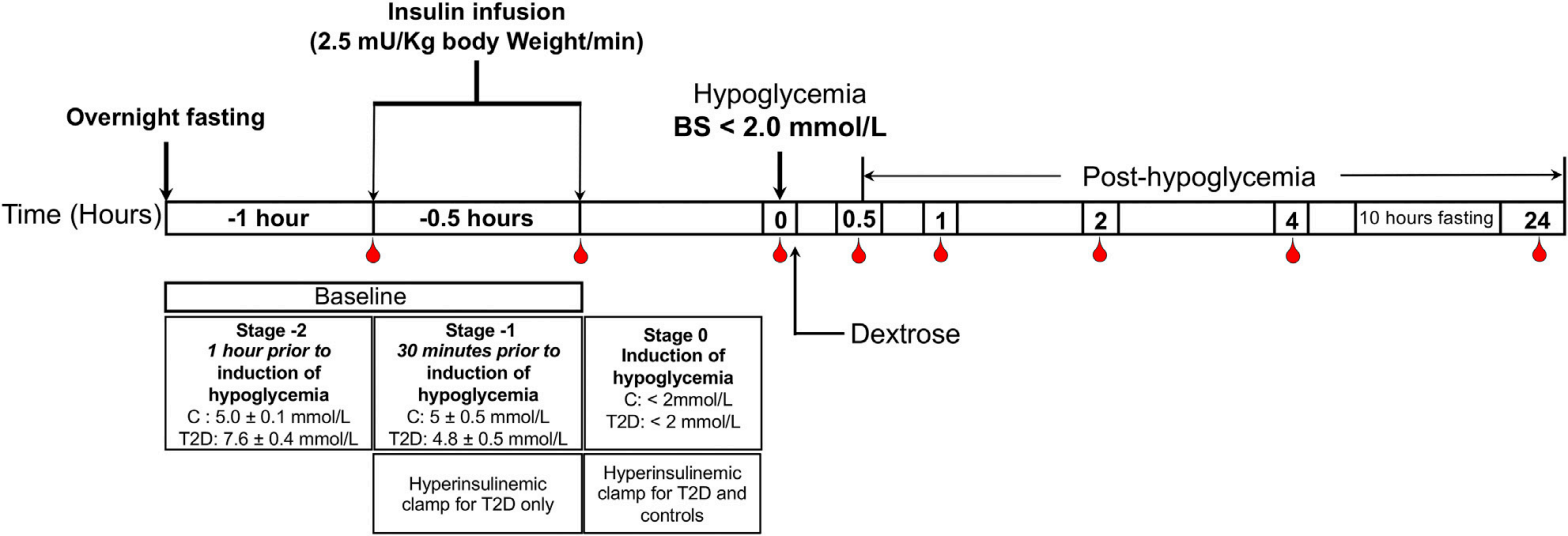
Schematic diagram of the insulin clamp study. The schematic indicates the intervention and blood sampling time points.

The hyperinsulinemic clamp was performed as previously reported (Kahal et al., 2020); all patients underwent a 10-h fast prior to the clamp. T2D: baseline glucose 7.6 ± 0.4 mmol/L (136.8 ± 7.2 mg/dl), reduced to 4.5 ± 0.07 mmol/L (81 ± 1.2 mg/dl) for 1-h. Controls: 4.9 ± 0.1 mmol/L (88.2 ± 1.8 mg/dl), following which the blood glucose was reduced in both control and T2D cohorts to <2.0 mmol/L (36 mg/dl) that was then reversed with intravenous glucose (150 ml of 10% dextrose) (Moin et al., 2021) (Figure 1).

### Biochemical Markers

Blood samples were centriguged (2000 g for 15-min at 4°C) and, within 15-min of collection, aliquots were stored at −80°C awaiting batch analysis. Using a Beckman AU 5800 analyser (Beckman-Coulter, High Wycombe, United Kingdom), enzymatic methods were used to determine fasting plasma glucose (FPG), total cholesterol, triglycerides, and high-density lipoprotein (HDL) cholesterol levels.

### SOMA-Scan Assay

Slow Off-rate Modified Aptamer (SOMA)-scan plasma protein measurement, as previously described (Kahal et al., 2020), was used to determine a panel of proteins purported to represent biomarkers of AIS: platelet basic protein (PPBP), thrombospondin-1 (THBS1), cadherin-1 (CDH1), and lymphatic vessel endothelial hyaluronic acid receptor 1 (LYVE1). SPP2, IGF2, and APOC4-APOC2 were not available in SOMAscan.

### Statistical Analysis

No studies showing changes in biomarker proteins of AIS in response to hypoglycaemia are available upon which a power calculation could be based. Birkett and Day (Birkett and Day, 1994) reviewed pilot study sample size and concluded that, to estimate effect size and variability, 20 degrees-of-freedom as a minimum was required. Hence, for this pilot study, a minimum of 20 patients per group was required. Visual evaluation of data trends for each parameter was undertaken; non-parametric tests were used for non-normal data as determined by the Kolmogorov-Smirnov Test. At each timepoint, the T2D and control groups were compared using Student’s t-test, with significance set at the level of *p* < 0.05. Within-group comparisons (changes from baseline, and from hypoglycemia, to each subsequent timepoint) were assessed with Student’s t-test. No adjustment for baseline covariates was made as the sample size was too small. Graphpad Prism (San Diego, CA, United States) was used for statistical analyses.

With regards to the proteomic analysis, an intercept-free general linear model was fitted as a function of a subgroup (i.e., condition:timepoint); patient ID was taken as a random effect using R package limma. Thereafter, the *p* value was calculated for two contrasts: baseline to hypoglycemia (for both the T2D and control cohorts), and false discovery rate (FDR) corrected at a significance of <0.05 as the cutoff.

## Results

Baseline demographic and biochemical data is shown in Table 1. Age was comparable between T2D (*n* = 23) and control (*n* = 23) subjects (*p* = ns); T2D had an elevated BMI (*p* = 0.0012) with duration of diabetes 4.5 ± 2.9 years. Systolic and diastolic blood pressure was elevated in the T2D cohort (*p* = 0.001 and *p* = 0.003, respectively), as was HbA1c (*p* < 0.0001).

**TABLE 1:** Study participant demographic and biochemical data. Data is presented as Mean ± 1 SD (Median).

Baseline	Type 2 diabetes (*n* = 23)	Controls (*n* = 23)	*p*-value	Statistically significant
Age (years)	64 ± 8 (66)	60 ± 10 (63)	0.15	No
Sex (M/F)	12/11	11/12	0.77	No
BMI (kg/m^2^)	32 ± 4 (32)	28 ± 3(27)	0.001	Yes
Systolic BP (mmHg)	132 ± 8 (130)	122 ± 8 (122)	0.0002	Yes
Diastolic BP (mmHg)	81 ± 7 (80)	75 ± 6 (75)	0.003	Yes
Duration of diabetes (years)	4.5 ± 2.2 (5.0)	N/A		N/A
HbA1c (mmol/mol)	51.2 ± 11.4 (50.0)	37.2 ± 2.2 (37.0)	<0.0001	Yes
HbA1c (%)	6.8 ± 1.0 (6.7)	5.6 ± 0.2 (5.5)	<0.0001	Yes
Total cholesterol (mmol/L)	4.2 ± 1.0 (4.1)	4.8 ± 0.67 (4.9)	0.02	Yes
Triglyceride (mmol/L)	1.7 ± 0.7 (1.5)	1.34 ± 0.6 (1.3)	0.06	No
HDL-cholesterol (mmol/L)	1.1 ± 0.3 (1.1)	1.5 ± 0.4 (1.4)	0.002	Yes
LDL-cholesterol (mmol/L)	2.27 ± 0.8 (2.1)	2.7 ± 0.7 (2.8)	0.06	No
CRP (mg/L)	3.0 ± 2.7 (1.9)	5.1 ± 10.3 (2.1)	0.33	No

BMI, body mass index; BP, blood pressure; HDL-cholesterol, High density lipoprotein cholesterol; LDL-cholesterol, Low density lipoprotein cholesterol; CRP, C-reactive protein; HbA1c, Haemoglobin A1c; N/A, not applicable.

**FIGURE 2:**
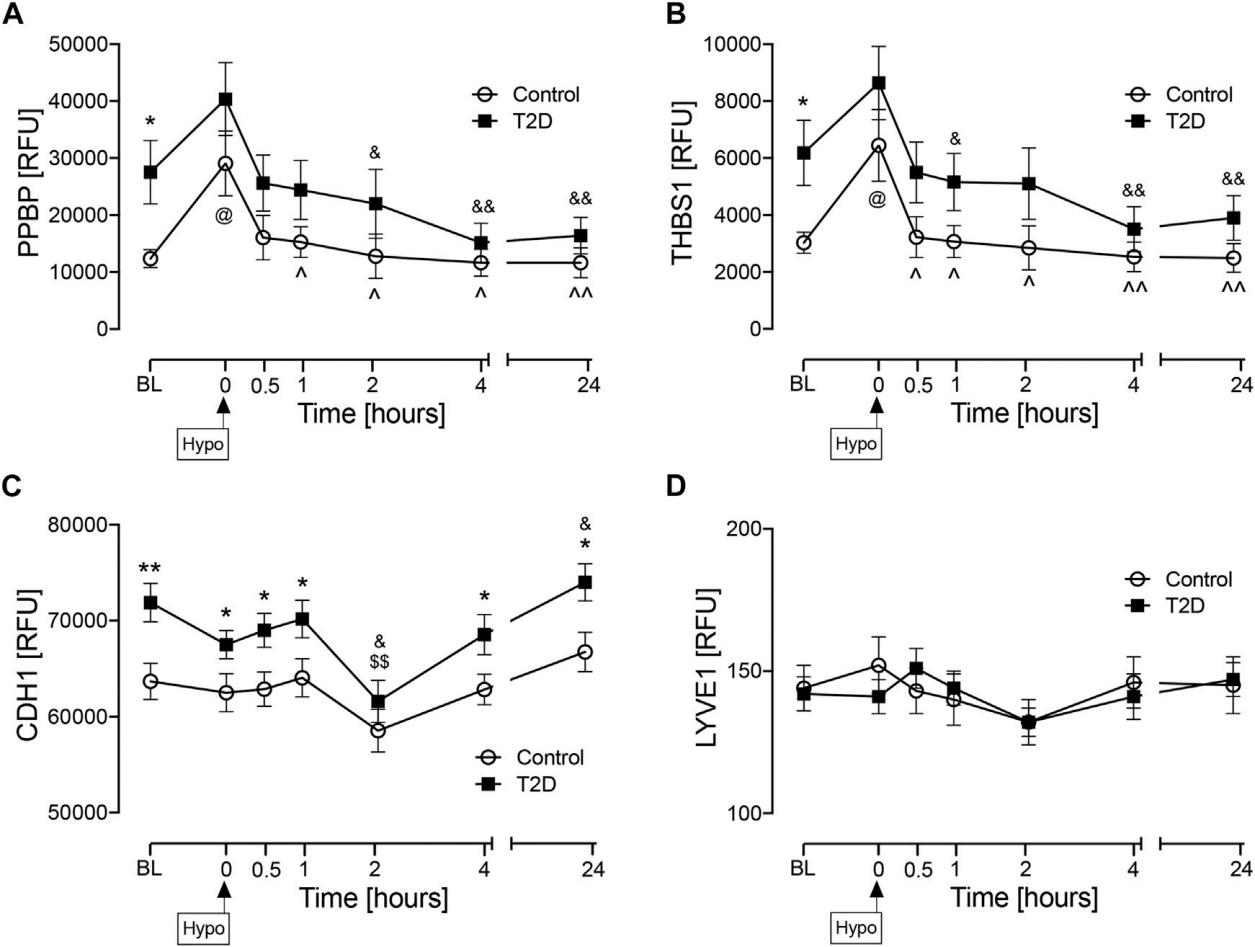
Circulatory levels of endothelial cell marker proteins at baseline, at hypoglycemia and post-hypoglycemia timepoints in T2D and control subjects. Blood sampling was performed at baseline (BL), at hypoglycemia (0 min) and post-hypoglycemia (30 min, 1-h, 2-h, 4-h, and 24-h) for controls (white circles) and for T2D (black squares). At baseline (BL), blood sugar (BS) was 7.5 ± 0.4 mmol/L (for T2D) and 5.0 ± 0.1 mmol/L (for control, C). At the point of hypoglycemia, blood sugar (BS) was 2.0 ± 0.03 mmol/L (for T2D) and 1.8 ± 0.05 mmol/L (for control). Proteomic (Somalogic) analysis of proteins was undertaken for platelet basic protein (PPBP) **(A)**, thrombospondin-1 (THBS1) **(B)**, cadherin-1 (CDH1) **(C)** and lymphatic vessel endothelial hyaluronic acid receptor 1 (LYVE1) **(D)**. Statistics: T2D *vs.* control: *p < 0.05, **p < 0.01; T2D BL *vs.* subsequent timepoints: $*p* < 0.05, $$*p* < 0.01; T2D hypoglycemia vs subsequent timepoints: &*p* < 0.05, &&*p* < 0.01; Control BL *vs.* subsequent timepoints: @*p* < 0.05; Control hypoglycemia *vs.* subsequent timepoints: ^*p* < 0.05. ^^*p* < 0.01. RFU, relative fluorescent units.

Plasma protein levels are shown in Figure 2. At baseline (BL), PPBP (*p* < 0.05) (Figure 2A), THBS1 (*p* < 0.05) (Figure 2B), and CDH1 (*p* < 0.01) (Figure 2C) were elevated in T2D whilst there was no difference between controls and T2D subjects at BL for LYVE1 (Figure 2D). Levels of LYVE1 remained unchanged throughout the study timecourse. PPBP and THBS1 showed very similar patterns, tending to increase at hypoglycemia in both T2D and controls, though only reaching significance in controls (*p* < 0.05, control BL versus control hypo) and returning to BL levels in both T2D and control subjects in the post-hypoglycemia follow-up period. CDH1 levels were higher in T2D at BL, at hypoglycemia and in the initial post-hypoglycemic phase until 2-h when levels decreased significantly in T2D, thereafter reverting to BL; in controls, CDH1 levels remained steady throughout the experimental timecourse.

### Additional Markers of Large Vessel Obstruction

**FIGURE 3:**
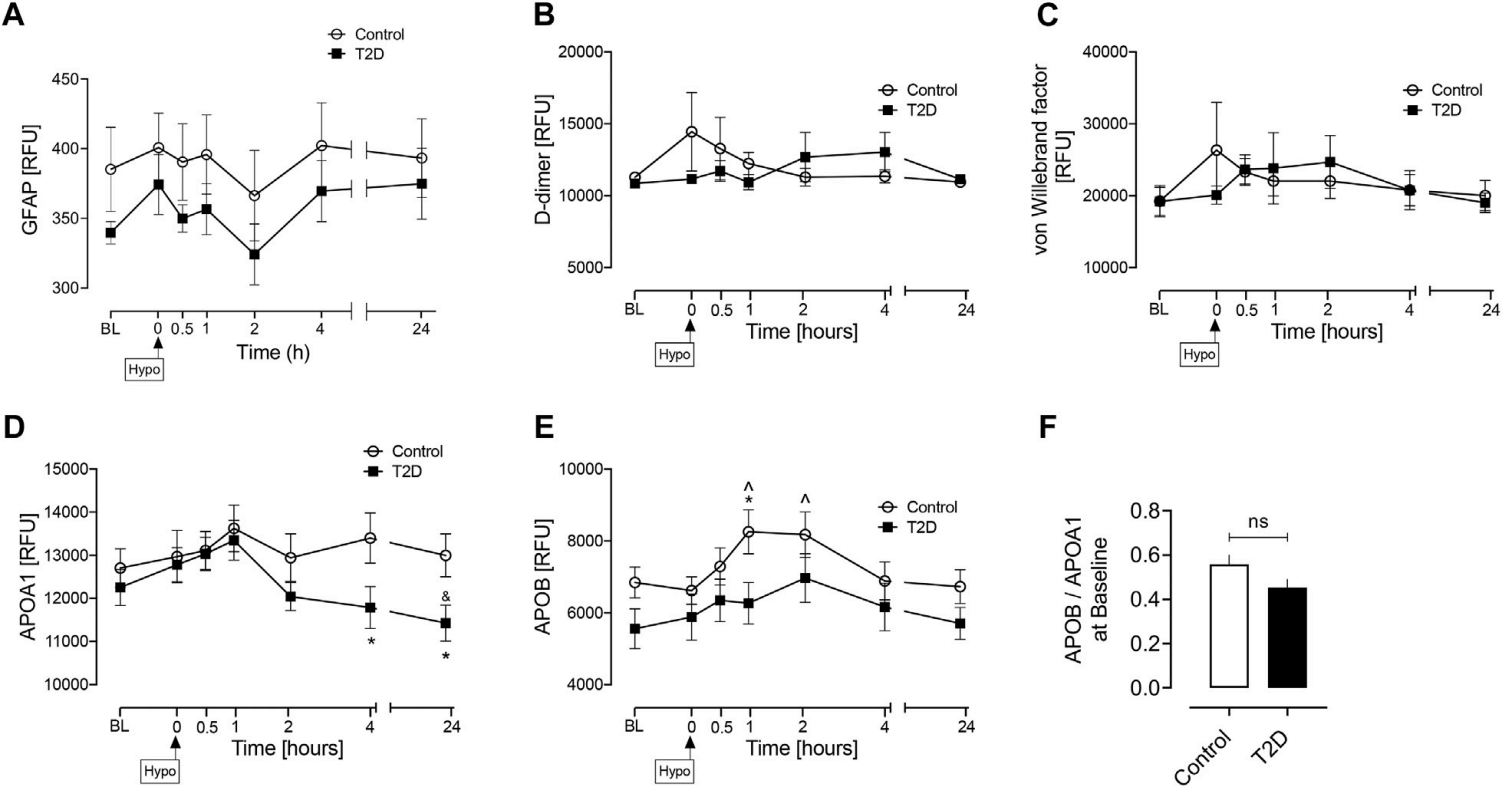
Circulatory levels of previously reported AIS due to LVO biomarkers at baseline, at hypoglycemia, and post-hypoglycemia timepoints in T2D and control subjects. Blood sampling was performed at baseline (BL), at hypoglycemia (0 min) and post-hypoglycemia (30 min, 1-h, 2-h, 4-h, and 24-h) for controls (white circles) and for T2D (black squares). Proteomic (Somalogic) analysis of proteins was undertaken for Glial fibrillary acidic protein, GFAP **(A)** D-dimer **(B)** von Willebrand factor, vWF **(C)** Apolipoprotein A1, APOA1 **(D)** Apolipoprotein B, APOB, **(E)** the ratio of basal levels of apolipoprotein B and apolipoprotein A1 (APOB/APOA1), **(F)**. Statistics: **(A–E)** T2D vs control: *p < 0.05, **p < 0.01; T2D BL *vs.* subsequent timepoints: $ *p* < 0.05, $$ *p* < 0.01; T2D hypoglycemia *vs.* subsequent timepoints: &*p* < 0.05, &&*p* < 0.01; Control BL *vs.* subsequent timepoints: @*p* < 0.05; Control hypoglycemia *vs.* subsequent timepoints: ^*p* < 0.05. ^^*p* < 0.01; **(F)** Data presented in the bar graph as mean ± SEM; ns, not significant.

As a subsequent analysis, baseline values were determined for those additional markers that have been reported in the literature as potential biomarkers of AIS due to LVO in acute hospitalized patients, namely Glial fibrillary acidic protein, GFAP; D-dimer; von Willebrand Factor, vWF; Apolipoprotein A1, APOA1; Apolipoprotein B, APOB along with the ratio of baseline levels of apolipoprotein B to apolipoprotein A1 (APOB/APOA1) (As et al., 2013; Katsanos et al., 2017; Sato et al., 2020; Baez et al., 2021; Dagonnier et al., 2021; Gaude et al., 2021; Ramos-Pachón et al., 2021); baseline levels of these proteins were not different between controls and T2D (Figure 3).

## Discussion

Baseline elevation of PPBP, THBS1, and CDH1 was surprising and is in accord with the fact that T2D patients have an enhanced AIS risk. The changes in the levels following hypoglycemic stress may also be indicative of increased risk of AIS, and exposure to hypoglycemia has been suggested to be associated with cardiovascular disease and stroke (Smith et al., 2018). This suggests that prospective studies in those patients with elevated levels should be undertaken to see if this translates into increased clinical risk that could be prophylactically addressed. However, it should be noted that the differences between T2D and controls were only approximately 10% and therefore, whilst statistically significant, it needs to be determined whether this is clinically significant.

Whilst PPBP was reported to be elevated in AIS, it appeared not to have any predictive value (Qin et al., 2019) and, in this study, whether its elevation in T2D is a biomarker of AIS needs clarifying.

As noted above, THBS1was shown to be elevated in AIS and had positive predictive value at 3-month prognosis (Qin et al., 2019); however, it remains unclear whether its elevation in T2D is a biomarker of AIS or whether it is indeed being protective. Similarly, LYVE1 was elevated in AIS and had positive predictive value at 3-month prognosis (Qin et al., 2019), though its levels did not differ in this study. CDH1 was reported to be downregulated in AIS (Qin et al., 2019); however, here, its levels remained higher in T2D at all timepoints, perhaps reflecting inflammation, with a marked decrease at 2-h that may have been due to the impact of hypoglycemia on the BBB.

Other proteins, such as Glial fibrillary acidic protein [GFAP], D-dimer, von Willebrand Factor [vWF], Apolipoprotein A1 [APOA1], and Apolipoprotein B [APOB], along with the ratio of baseline levels of apolipoprotein B to apolipoprotein A1 [APOB/APOA1 ratio], have been purported to be potential biomarkers for AIS due to LVO (As et al., 2013; Katsanos et al., 2017; Sato et al., 2020; Baez et al., 2021; Dagonnier et al., 2021; Gaude et al., 2021; Ramos-Pachón et al., 2021). However, these potential biomarkers have been reported in cohorts of acutely ill, hospitalized patients; here, we studied an ambulatory T2D cohort who had a relatively short duration of disease (4.5 ± 2.2 years) with no diabetic complications and therefore have only a minimally higher risk of AIS relative to their non-diabetic counterparts. Therefore, this outpatient cohort is lower risk and not comparable to the severe, high risk inpatient cohorts reported, and therefore it was not surprising that there were no differences between controls and T2D in this non-acute setting.

Short disease duration without diabetes complications perhaps also helps to explain why the baseline elevations of PPBP, THBS1, and CDH1 were modest in this T2D population, though may additionally indicate that they are more sensitive markers of AIS due to LVO.

Strengths of this study include that T2D subjects had a short disease duration and were on minimal anti-diabetic therapy. The main study limitation is the small study numbers in this proteomic exploratory study although, despite this, clear differences were seen between T2D and control cohorts. Although the T2D subjects were more obese, this should not have altered the expression of these proteins in response to the hypoglycemic insult. All subjects were Caucasian, and therefore these results may not be generalizable to other ethnic groups. One other notable feature was that the systolic and diastolic blood pressures were higher in the diabetes population, though not hypertensive, and whilst the diastolic blood pressure was lower in the control population this was not abnormally low (diastolic hypotension being defined as less than 60 mmHg); none of the subjects had postural symptoms. In a community dwelling cohort of 3544 subjects aged 60 and older, 20% had a diastolic blood pressure less than 80 mmHg and 50% had a diastolic blood pressure between 80 and 89 mmHg, suggesting that these values of 81 mmHg in T2D and 75 mmgHg in controls in subjects without hypotensive symptoms are not abnormally low (Klein et al., 2013), though the median values in this study differ to that report (Klein et al., 2013), likely due to the populations being different between the two studies. In this same study of 3544 subjects, the systolic blood pressure was 139 mmHg or less in 582 subjects (16%) (Klein et al., 2013), again suggesting differences between the populations. The higher systolic and diastolic blood pressures in diabetes are in accord with data showing that those with diabetes may have a higher blood pressure than those without diabetes (Wright et al., 2020) and with a much higher incidence of progressing to frank hypertension.

In conclusion, patients with T2D showed elevated levels of PPBP, THBS1, and CDH1, circulatory proteins that have been suggested to be biomarkers of AIS due to LVO, and therefore may identify a cohort of T2D patients at increased risk of AIS. This data suggests that prospective studies on this group of subjects should be undertaken to see if this reflects future clinical risk and would therefore impact clinical practice. Further prospective studies could also explore whether newer anti-diabetic medications, such as glucagon-like peptide-1 receptor agonists (GLP1-RAs) and sodium-glucose cotransporter 2 inhibitors (SGLT2is), that can lower the risk of cardiovascular disease, might have an impact upon the circulating levels of the biomarkers studied here.

## Data Availability

The raw data supporting the conclusion of this article will be made available by the authors, without undue reservation.
